# Therapeutic Effect Enhancement by Dual-Bias High-Voltage Circuit of Transmit Amplifier for Immersion Ultrasound Transducer Applications

**DOI:** 10.3390/s18124210

**Published:** 2018-11-30

**Authors:** Hojong Choi, Se-woon Choe

**Affiliations:** Department of Medical IT Convergence Engineering, Kumoh National Institute of Technology, Gumi-Daero 350–27, Gumi 39253, Korea; hojongch@kumoh.ac.kr

**Keywords:** Dual-bias high-voltage circuit, transmit amplifier, immersion ultrasound transducer

## Abstract

The dual-bias high-voltage circuit of a transmit amplifier for immersion ultrasound transducer applications is proposed to enhance the therapeutic effect of human HeLa cells. High-voltage output signals generated from a transmit amplifier are typically preferable for immersion ultrasound transducers owing to their high sensitivity at the desired frequency. However, high-voltage output signals typically produce high-order harmonic distortions, thus triggering several unwanted high-order spectral signals in the ultrasound transducers. By reducing high-order harmonic distortions, we expect that improving the signal quality of excited pulses for immersion ultrasound transducers would be beneficial for the therapeutic effect on human cervical cancer HeLa cell suppression. Therefore, an additional bias circuit is developed to merge with the original bias circuit for transmit amplifier to control the harmonic distortions of the immersion ultrasonic transducer. To properly select the components of dual-bias high-voltage circuit, we need to calculate and measure the DC bias voltages of the transmit amplifier with and without dual-bias high-voltage circuit for different period of the time for therapeutic applications. To evaluate the performances of the developed circuit, pulse-echo measurements using a transmit amplifier with or without dual-bias high-voltage circuit were obtained. The measured second, third, and fourth harmonic distortions of the echo signals when using the transmit amplifier with dual-bias high-voltage circuit at 10 V DC bias voltage are lower than those when using the transmit amplifier only. Subsequently, the therapeutic effects using the enhanced performances of the transmit amplifier with dual-bias high-voltage circuit were verified and compared with those using the performances of the transmit amplifier by comparison of quantitative changes in HeLa cell concentrations. The control group without any ultrasonic induction increased the cell density up to about 100% on Day4, however the experimental groups with ultrasonic induction (TA = 91.2 ± 0.8%, TA+Dual-bias high-voltage circuit (0.8 V) = 78.8 ± 1.7% and TA+Dual-bias high-voltage circuit (10 V) = 66.3 ± 1.1%) showed statistically significant cell density changes compared to the control group. We confirmed that the therapeutic effect from using the dual-bias high-voltage circuit is improved. Therefore, it can be a potential candidate to improve the therapeutic effect of HeLa cells.

## 1. Introduction

Ultrasound systems have been widely used for diagnostic and therapeutic applications [1,2,3,4]. They are typically composed of transmitters, transducers, and receivers. Immersion ultrasound transducers are typically piezoelectric or capacitive transducers that are triggered by the voltage or current outputs of the transmitters [5,6]. They could convert the electrical powers to ultrasound powers or vice versa [7]. For therapeutic applications in ultrasound systems, transmit amplifiers in the transmitters are the most dominant electronic components that trigger immersion ultrasound transducers directly to propagate the ultrasound waves into the desired targets [8]. Therefore, the performance improvements of transmit amplifiers are one of the most critical works in the system design for therapeutic applications. The transmit amplifiers typically generate high-voltage output in ultrasound applications. Therefore, high output excitation voltages from the transmit amplifier are desirable to generate a high ultrasound powers from the immersion ultrasound transducers. As the input power in the transmit amplifiers increases, the output powers of the transmit amplifiers increase up to saturation [9]. However, transmit amplifiers are supposed to exhibit high harmonic distortions when generating high-voltage output signals, because the capacitances and inductances that are composed of transmit amplifiers exhibit nonlinear characteristics with respect to the excitation of the output voltages [10,11]. 

Uni-polar and bipolar pulsers have been widely used to trigger the ultrasound transducers for ultrasound applications [12]. However, they generate high even-order harmonic distortion components. The level-shifter circuit in the pulsers could generate unwanted noise signals from the power supply. The push-pull type transmit amplifiers using transformers could possible solution to reduce even-order harmonics [13]. However, transformers could generate the unwanted ring-down of the high-voltage outputs. An electrical filter circuit could be a solution to reduce the high-order harmonic distortions of the high-voltage output signals because the electrical filter is used to filter out high-order harmonic distortions in certain frequency ranges [9]. However, it is changeling to reduce intermodulation spectrum products of the adjacent output signals combined with the noise signals at certain voltage ranges produced by the transmit amplifiers [14,15]. Additionally, the electrical filter located after the transmit amplifiers that are typically composed of inductance and capacitances may introduce additional harmonic distortion components to the immersion ultrasound transducers. This could generate additional unwanted ring-downs of high-voltage output pulses, thus increasing harmonic distortions. To minimize this nonlinear characteristics of the capacitances and inductances especially in certain high-voltage output signals, the appropriate bias voltages to provide the primary transistors in transmit amplifiers could be a possible solution [16]. This similar approach has been used widely in radio-frequency devices [17,18]. The unwanted heat caused by high-voltage operations for the therapeutic applications could also affect the DC bias voltages of the transmit amplifier, thus possibly changing DC bias voltages from linear to non-linear operations [13,16]. The stable DC bias voltages for linear operation are very important because the transmit amplifier directly triggers the immersion ultrasound transducers for therapeutic applications [19]. Otherwise, changed DC bias voltage in the transmit amplifier could reduce the output voltages, thus obtaining a lower sensitivity of the ultrasound transducers [10]. The regulator needs to provide constant DC bias voltages against variances in DC power supply for transmit amplifier [20]. However, unwanted high temperature caused by high-voltage operations affects the resistance values of the active and passive components, thus affecting voltage variances in the regulator [21,22]. Therefore, the transmit amplifier operations could be switched from linear to non-linear operations accordingly.

**Figure 1:**
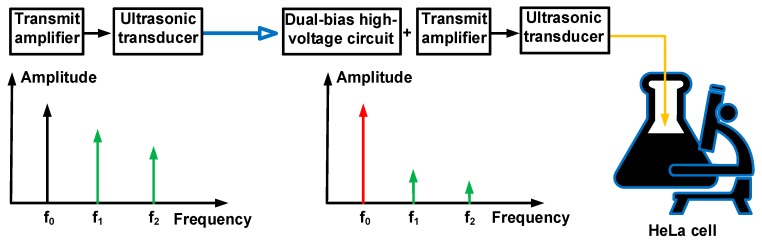
Concept of dual-bias high-voltage circuit and transmit amplifier for HeLa cell therapeutic effects.

Each HeLa cell could be induced differently by certain operating frequency ranges of the triggering devices [23]. The cell behaviors could be affected owing to the transmit amplifiers that produce several harmonic signals. Therefore, we developed a bias circuit to improve the transmit amplifier performances to enhance the therapeutic effects on the HeLa cells, as the sensitivity at the operating frequency is more strengthened compared to that at the second, third and higher harmonic frequencies (f_1_, f_2_, and f_n_, respectively), as illustrated in Figure 1. We propose a transmit amplifier with bias-circuit circuit triggering immersion ultrasound transducers for improving the therapeutic effects of the cells. Section 2 describes the design, analysis, and implementation of the dual-bias high-voltage circuit with the transmit amplifier. Section 3 describes the measured performances of the transmit amplifier with or without the dual-bias high-voltage circuit, pulse-echo and HeLa cell concentrations, as well as the comparison when using the transmit amplifier with or without the dual-bias high-voltage circuit. Section 4 presents the conclusion of the paper.

## 2. Materials and Methods

### 2.1. Design of Dual-Bias High-Voltage Circuit

**Figure 2:**
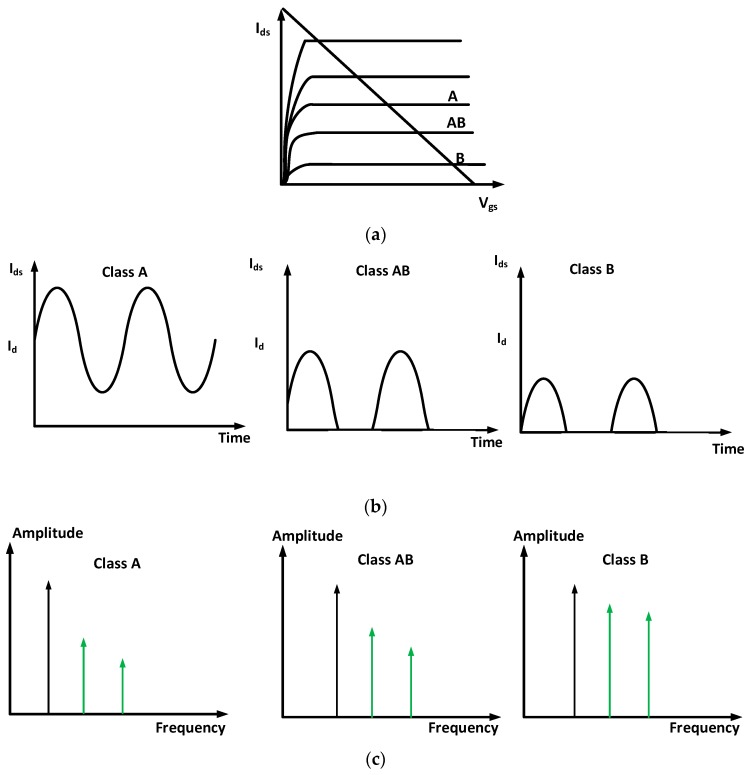
(**a**) The DC analysis and (**b**) drain-to-source current (I_ds_), and (**c**) spectrum data comparison of the transmit amplifier for Class A, AB, and B operations.

Figure 2 shows the characteristics of the transmit amplifier working in Class A, AB, and B operations in DC, time, and frequency domains. As shown in Figure 2a, the transmit amplifier is working is for linear (Class A) operation. However, the DC bias points for therapeutic applications could be affected by unwanted heat caused by high-voltage output signals [9,15]. In our experiment, to improve the therapeutic effects of HeLa cells, the transmit amplifier needs to be working more than 3-h. The DC bias point could be changed from linear (Class A) to non-linear operation (Class AB and B) points [13]. That could distort the high-voltage output signals of the transmit amplifier [24]. Therefore, we need to provide additional bias circuits to stabilize DC operating points for long-term operations in the therapeutic instruments. As shown in Figure 2b,c the Class A type operations do not suffer the signal distortions compared to Class AB and B. It is inaccurate to measure the current in the time domain such that we need to measure the spectrum data in the frequency domain to estimate how much signal distortions could be measured for Class A, AB, and B operations [11,25].

**Figure 3:**
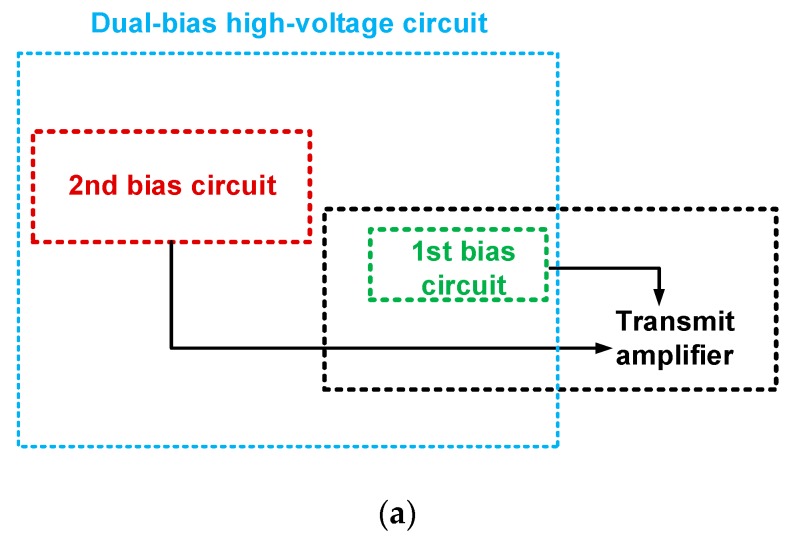

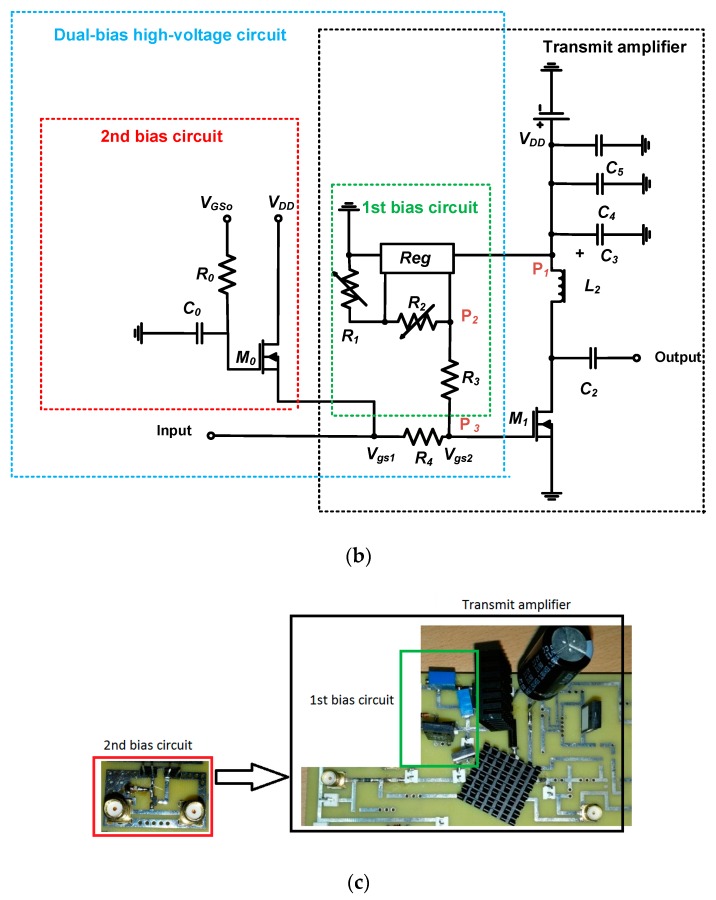
(**a**) Block diagram, (**b**) schematic diagram, and (**c**) PCB of the transmit amplifier integrated with dual-bias high-voltage circuit.

A transmit amplifier with dual-bias high-voltage circuit were implemented on a two-layer printed circuit board (PCB), respectively. Figure 3 shows the block diagram, schematic diagram and PCB of the transmit amplifier with dual-bias high-voltage circuit. In the transmit amplifier (Figure 2a), a 220 μF electrostatic capacitor (*C*_3_) and 1 μF and 0.1 μF by-pass capacitors (*C*_4_ and *C*_5_, respectively) were used to minimize the noises from the power supply for the transistor (*M*_1_). Further, 2 kΩ variable resistors (*R*_1_ and *R*_2_) and a resistor (*R*_3_) were used to provide the proper gate bias voltages for the primary transistor (*M*_1_). A 0 Ω surface-mount resistor (*R*_4_) was used to be connected between the bias voltages (V_gs1_ and V_gs2_) of 1st bias circuit and 2nd bias circuit due to empty space in the PCB.

In the dual-bias high-voltage circuit (Figure 3b), a high-voltage power transistor (*M*_0_) was used to occupy large voltage variances up to the DC power supply voltage (*V*_DD_), as the transmit amplifier required for large high voltage DC voltages for therapeutic applications. Depending on the gate and drain DC voltages (*V*_GS0_ and *V*_DD_), different DC bias voltages can be applied to the gate of the transmit amplifier. In the 2nd bias circuit, a large bypass capacitor (*C*_0_) was added to filter out the possible noises from the power supply, and a resistor (*R*_0_) was added to control the electrical impedance values with a high-voltage transistor (*M*_0_) and avoid abrupt noise signals in the 2nd bias circuit. Therefore, the 2nd bias circuit functions work as a variable resistor at certain high-voltages. This could be desirable for long-term operations which generate unwanted heat for transmit amplifier. Figure 2c shows the fabricated PCB of transmit amplifier with dual-bias high-voltage circuit (1st bias circuit and 2nd bias circuit). The transmit amplifier and dual-bias high-voltage circuit were implemented as a PCB fabricated from ExpressPCB, LLC (Mulino, OR, USA). The heat-sink was attached on the top and bottom of the transistor [26]. However, the heat-sink cannot completely stabilize the performances of the transmit amplifier for therapeutic applications.

**Figure 4:**
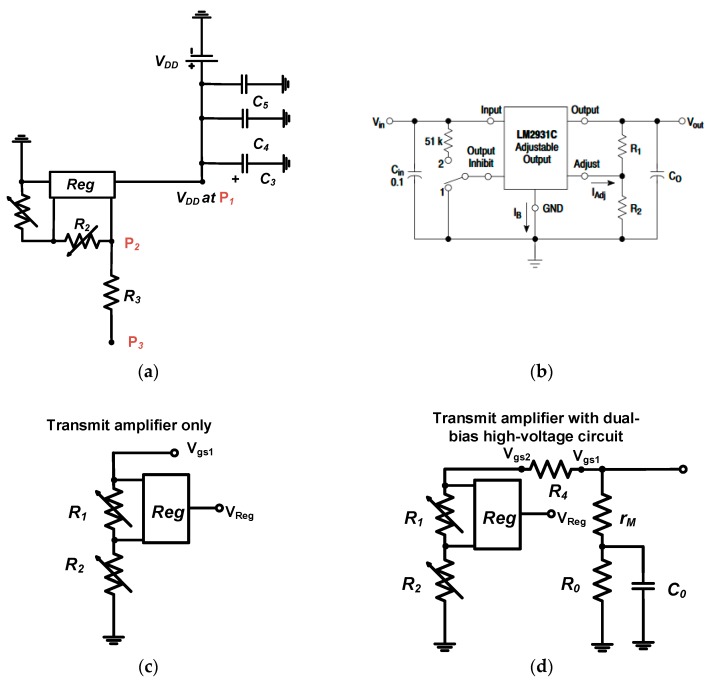
(**a**) Schematic diagram of gate-source voltage of the transmit amplifier and (**b**) voltage regulator (LM2931C); Equivalent circuit models of gate-source DC bias voltage of (**c**) transmit amplifier only (**d**) and transmit amplifier with dual-bias high-voltage circuit.

As shown in Figure 3b, voltage regulator (*Reg*) is the electronic component to provide certain DC voltages. The voltage regulator (LM2931C, On-semiconductor, Phoenix, AZ, USA) was used to provide the DC voltages to the gate of the primary transistor (M_1_). Figure 4a shows the part of the schematic diagram of the gate-source voltage of the transmit amplifier. The DC voltage at P_1_ point is same as V_DD_ in the power supply. A DC voltage at point P_2_ is changed after the voltage regulator. The DC bias voltage at P_2_ and P_3_ points are supposed to be same. Figure 4b shows the schematic of the voltage regulator (LM2931C) with two resistors [27]. The output voltage depends on the input voltage with two resistors.

As shown in Figure 4c, the gate-source voltage of the transistor (V*_gs_*_1_) depends on the variable resistors (*R*_1_ and *R*_2_) and regulator voltage (V*_Reg_*) since adjustable current (*I_Adj_*) generated from voltage regulator is typically small and large resistor (*R*_3_) is used between the *P*_2_ and *P*_3_ such that it can be expressed as in Equation (1).

(1) $$V_{gs1}=V_{Reg}\cdot \left[\frac{R_{1}+R_{2}}{R_{2}}\right]+I_{Adj}R_{2}\approx V_{Reg}\cdot \left[1+\frac{R_{1}}{R_{2}}\right]$$

where V*_Reg_* and *I_Adj_* are the reference voltage and adjustable current of the voltage regulator.

The calculated gate-source voltage of the transistor (V*_gs_*_1_) is 4.9 V since the regulator voltage (V*_Reg_*) is 3.5 V. As shown in Figure 4c, the voltage regulator needs to utilize two variable resistors. Those resistor values could be highly affected by unwanted heat for long-term operations. Therefore, the gate-source voltages are likely to be varied depending on the resistance value variances. If the resistance value variances are large, the gate-source voltage could be affected directly even though the voltage regulator provides a constant DC voltage. Therefore, we developed the additional bias voltage circuit to stabilize the DC bias voltage of the transmit amplifier. 

Using the 2nd bias circuit (Figure 4d), the gate-source voltage of the transistor (*M*_1_) was affected by the variable resistors (*R*_1_ and *R*_2_) of the high-voltage transistor (*M*_0_) parallel with the transconductance, resistor, and capacitor. Therefore, it can be expressed as in Equation (2).

(2) $$V_{gs1}=V_{Reg}\cdot \left[\frac{2\pi f_{c}C_{0}R_{0}}{\sqrt{{\left(1+r_{M}+R_{0}+\frac{R_{1}}{R_{2}}\right)}^{2}+{\left(2\pi f_{c}C_{0}R_{0}\left(1+r_{M}+\frac{R_{1}}{R_{2}}\right)\right)}^{2}}}\right]$$

where *r_M_* is the transconductance of the transistor (*M*_0_) and *f*_c_ is the operating frequency of the transmit amplifier.

The calculated gate-source voltage of the transistor (*M*_1_) is 4.99 V since the transconductance is 10 Ω and operating frequency of the transmit amplifier is 1 MHz. As shown in Equation (2), the gate-source voltage can be changed by the resistance of the high-voltage transistor (*r_M_*) with a resistor (*R*_0_) and capacitor (*C*_0_) such that the gate-source voltage (V*_gs_*_1_) can be controlled by those components, thus providing different DC bias voltage at a certain operating frequency. 

**Figure 5:**
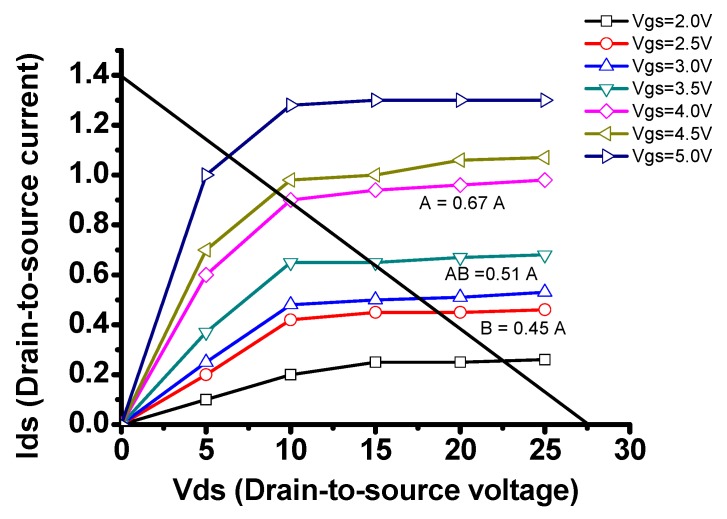
DC analysis points.

Even though we calculated the gate-source bias voltage (V*_gs_*_1_) when using the transmit amplifier with and without dual-bias high-voltage circuit, the equivalent circuit models of the transmit amplifier are inaccurate to predict the DC analysis caused by unwanted heat due to high-voltage operations [28,29]. Therefore, we need to obtain the DC analysis. Figure 5 shows the DC analysis points for transmit amplifier with respect to the different gate-to-source voltages.

Table 1 shows DC gate-source bias voltage (V*_gs_*_1_) of the isolated transmit amplifier and transmit amplifier integrated with dual-bias high-voltage circuit at 1 min, 1 h, 2 h, and 3 h since calculated DC bias gate-source voltage does not contain temperature effects. As shown in Table 1, the gate-source voltages of the transmit amplifier moved from 4.0 V to 2.75 V which represents that the transmit amplifier moves Class A to Class AB and B operations. However, the gate-source voltages of the transmit amplifier integrated with dual-bias high-voltage circuit slightly reduced from 4.0 V to 3.92 V which still remains Class A operation. While the DC gate-source bias voltage of the isolated transmit amplifier is mainly provided from the regulator with two variable resistors, the DC bias voltage of the transmit amplifier integrated with dual-bias high-voltage circuit is provided by the regulator with two variable resistors and high-voltage transistor in the 2nd bias circuit together.

Different DC bias voltages could change the performances of the transmit amplifier such that we will further measure the power gain deviations and harmonic distortions in the spectrum domain in Section 3.1.

### 2.2. HeLa Cell Preparation

Human cervical carcinoma HeLa cells (Korean Cell Line Bank, Seoul, Korea) were cultured in high- glucose Dulbecco’s Modified Eagle Medium containing 10% Fetal Bovine Serum and 1% penicillin streptomycin. The prepared cells were incubated at 37 °C in a humidified benchtop incubator with 5% CO_2_. The cells were trypsinized when the growing cells reached confluence at 70%, washed thrice with phosphate-buffered saline and resuspended to yield an approximate concentration of $1\times 10^{6}$ cells/mL in the cell culture dish. When the cell confluence reached 30% to 40%, an ultrasound stimulus was induced and subsequently counted as day 0. The ambient temperature was adjusted to 26 °C to avoid thermal damages on the HeLa cells. To induce an ultrasound signal on the same cells in the culture dish during days 0–4, a customized immersion ultrasound transducer holder was designed and printed using a three-dimensional printer (Cubicon 3DP-310F, High Vision System, Sungnam, Korea). A fixed immersion ultrasound transducer with the customized holder was positioned on the surface of the growth media in the cell culture dish, and its position was maintained during the experiment from day 0 to day 4. All samples were divided into four groups: control group (no ultrasonic induction, n = 6), TA group (ultrasonic induction with transmit amplifier, n = 6), TA+Dual-bias high-voltage circuit (ultrasonic induction with transmit amplifier and dual-bias high-voltage circuit at 0.8 V, n = 6) and TA+Dual-bias high-voltage circuit (ultrasonic induction with transmit amplifier and dual-bias high-voltage circuit at 10 V, n = 6). 

The ultrasonic signal from the immersion ultrasound transducer connected to the transmit amplifier only, and the transmit amplifier integrated with the dual-bias high-voltage circuit was induced daily for 30 min. The microscopic brightfield images of ultrasound signal focused area in the cell culture dish were acquired immediately after ultrasound signal induction, using an inverted fluorescent microscope (IX73-DP80, Olympus Corp., Tokyo, Japan). To quantify the cell number and area of the grown cells, image processing was performed using the Matlab software (MathWorks, Natick, MA, USA), and they were analized statistically using ANOVA with Scheffe’s post-hoc test. The cell density was calculated by dividing the pixel number of the HeLa cell grown area by the acquired microscopic pixel number. A *P* value of less than 0.05 was considered statistically significant compare to the control group.

## 3. Results and Discussion

### 3.1. Performances of Transmit Amplifier and Dual-Bias High-Voltage Circuit

**Figure 6:**
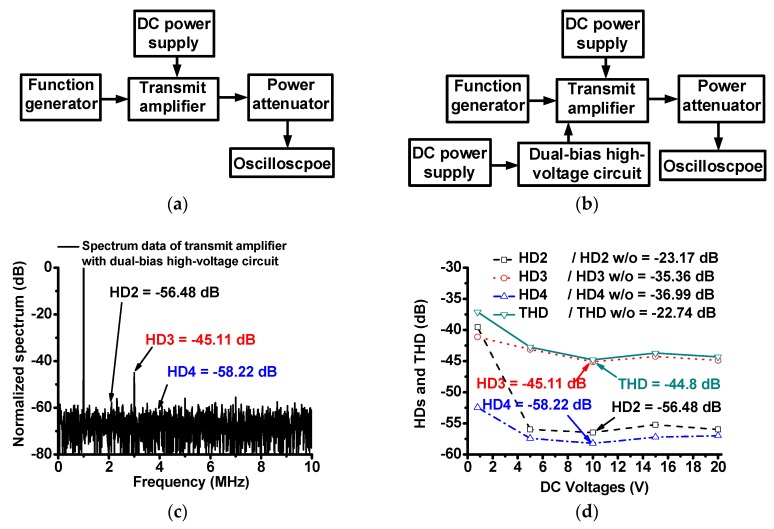
Block diagram of measurement system when using (**a**) transmit amplifier only and (**b**) transmit amplifier with dual-bias high-voltage circuit, (**c**) spectrum data of transmit amplifier with dual-bias high-voltage circuit at 10 V DC voltage, and (**d**) HD2, HD3, HD4 and THD values with and without dual-bias high-voltage circuit vs. 0.8 V, 5 V, 10 V, 15 V and 20 V DC voltages (graph with dash, dot, dash-dot and solid marks represent HD2, HD3, HD4, and THD, respectively, of the transmit amplifier with dual-bias high-voltage circuit). HD2 w/o, HD3 w/o, HD4 w/o, THD w/o represent the measured HD2, HD3, HD4, and THD of the output signals, respectively, when using transmit amplifier only.

Figure 6a,b shows the measurement setups of the transmit amplifier and high-voltage dual-bias circuit. As described in Figure 2, changed DC operating points in the non-linear operation could increase signal distortions of the transmit amplifier. Since the current measurement in the time domain is not accurate, we need to measure the harmonic signal distortion in the spectrum domain when using the transmit amplifier with and without dual-bias high-voltage circuit. The DC bias voltage ranges of the power transistor is from 0.8 V to 20 V DC on a 25 V drain-source voltage, as maximum gate-source voltage of the power transistor is between 0.8 V and 20 V. Further 1-MHz, 417 mV_p-p_, and 10-cycle sinusoidal pulses generated from a function generator (AFG3252, Tecktronics Inc., Beaverton, OR, USA) were sent to the designed transmit amplifier with and without dual-bias high-voltage circuit, with the DC bias voltage from a power supply (E3631A, Agilent Technologies, Santa Clara, CA, USA). Additionally, the spectrum data from an oscilloscope (MSOX4154A, Keysight Technology, Santa Clara, CA, USA) were plotted. Figure 6c shows the spectrum data when using transmit amplifier with dual-bias high-voltage circuit at 10 V DC voltage. 

Figure 6d shows the second, third, and fourth harmonic distortions (HD2, HD3, and HD4, respectively), and total harmonic distortion (THD) of the transmit amplifier with and without dual-bias high-voltage circuit. The measured HD2, HD3, HD4, and THD of the echo signals when using the transmit amplifier with dual-bias high-voltage circuit (HD2, HD3, HD4, and THD = –56.48 dB, –45.11 dB, −58.22 dB, and –44.8 dB at 10V DC voltage, respectively) was substantially lower than those when using the transmit amplifier only (HD2, HD3, HD4, and THD = –23.17 dB, –35.36 dB, –36.99 dB, and –22.74 dB, respectively). As shown in Figure 4d, all HD2, HD3, HD4, and THD values of the transmit amplifier with dual-bias high-voltage circuit were lower than those of the transmit amplifier only. Additionally, the HD2, HD3, HD4 and THD values of the transmit amplifier with dual-bias high-voltage circuit exhibit similar values (less than –2.5 dB) with respect to different DC bias voltages after 0.8 V DC. Therefore, the dual-bias high-voltage circuit can stabilize the performances of the transmit amplifier, thus reducing the harmonic distortions of the transmit amplifier. These measurement data represent that the dual-bias high-voltage circuit helps the transmit amplifier obtain stable DC bias voltages.

### 3.2. Pulse-Echo Measurement

**Figure 7:**
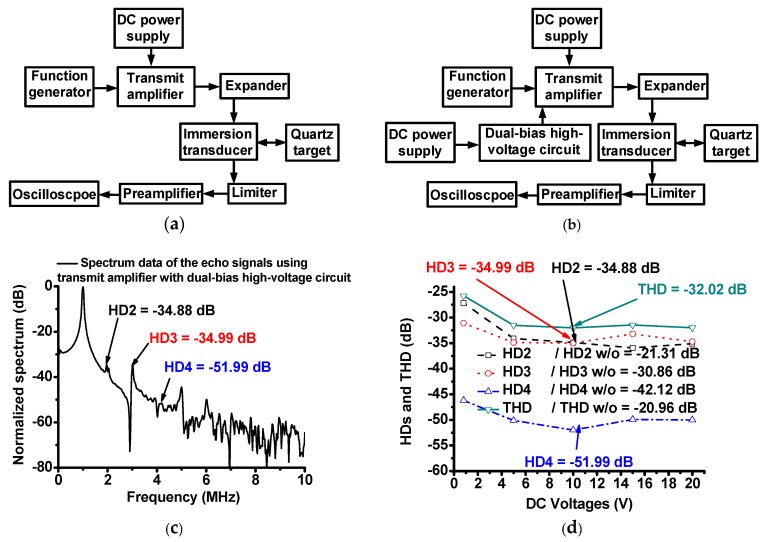
Block diagram of pulse-echo test measurement system when using (**a**) transmit amplifier only and (**b**) transmit amplifier with dual-bias high-voltage circuit, (**c**) spectrum data of echo signal generated by a 1 MHz immersion ultrasound transducer with transmit amplifier with dual-bias high-voltage circuit at 10 V DC bias voltage, and (**d**) HD2, HD3, HD4 and THD values with and without dual-bias high-voltage linearizer vs. 0.8 V, 5 V, 10 V, 15 V and 20 V DC bias voltages (graph with dash, dot, dash-dot and solid mark represent HD2, HD3, HD4, and THD of the transmit amplifier with dual-bias high-voltage circuit, respectively). HD2 w/o, HD3 w/o, HD4 w/o, THD w/o represent the measured HD2, HD3, HD4, and THD of the echo signals when using transmit amplifier only, respectively.

As shown in Figure 7a,b, the pulse-echo measurement is typical one-way measurement of the ultrasound transducer performances [30]. To confirm the capability of the dual-bias high-voltage circuit, the harmonic distortion values of the transmit amplifier with dual-bias high-voltage circuit were measured and compared. The transmit amplifier only and the transmit amplifier with the dual-bias high-voltage circuit with respect to the operating DC voltages were measured to obtain the operating conditions of the circuit. Additionally, 1-MHz and 10-cycle sinusoidal pulses generated from the transmit amplifier with and without dual-bias high-voltage circuit with the DC bias voltage from the power supply (E3631A) were transferred through an expander, and the received echo signals obtained from the immersion ultrasound transducer were passed through a limiter and preamplifier; subsequently, the spectrum data were displayed in the oscilloscope (MSOX4154A) from the quartz target. Figure 5c shows the HD2 and HD3 values of the 1-MHz immersion ultrasound transducer (V303, Olympus NDT. Inc., Waltham, MA, USA) triggered by the transmit amplifier with and without dual-bias high-voltage circuit at 10 V DC bias voltages. After 0.8 V DC bias voltages, the HD2, HD3, and THD values were reduced because the resistance values of the high-voltage transistor can be varied with respect to the applied voltage from the power supply. Therefore, we selected a certain DC voltage on the dual-bias high-voltage circuit for the HeLa cell experiments at 0.8 V and beyond 0.8 V.

The measured HD2, HD3, and HD4 of the echo signals when using the transmit amplifier with dual-bias high-voltage circuit (HD2, HD3, HD4, and THD = –34.88 dB, –34.99 dB, and –51.99 dB, respectively) were lower than those when using the transmit amplifier only (HD2, HD3, HD4, and THD = –21.31 dB, –30.86 dB, and –42.12 dB, respectively). Figure 5d shows a comparison of the measured HD2, HD3, HD4, and THD when using the transmit amplifier with and without dual-bias high-voltage circuit. All HD2, HD3, HD4, and THD values when using transmit amplifier with dual-bias high-voltage circuit (HD2, HD3, HD4, and THD = –34.88 dB, –34.99 dB, –51.99 dB, and –32.02 dB, respectively at 10 V DC bias voltage) were lower than those of the transmit amplifier only (HD2, HD3, HD4, and THD = –21.31 dB, –30.86 dB, –42.12 dB, and –20.96 dB, respectively). Additionally, the HD2, HD3, HD4 and THD values of the transmit amplifier with dual-bias high-voltage circuit were similar (less than –1.5 dB) with respect to the different DC bias voltages beyond 0.8 V DC. Therefore, we conclude that the dual-bias high-voltage circuit can stabilize the performances of the transmit amplifier with a wide DC bias voltage ranges by reducing the harmonic distortions of the transmit amplifier, thus generating reduced harmonic distortions of the echo signals. This can produce more clean signals at the desired operating frequency, thus possibly improving the performances of the treatment effects on the test cells.

### 3.3. HeLa Cell Experiments

**Figure 8:**
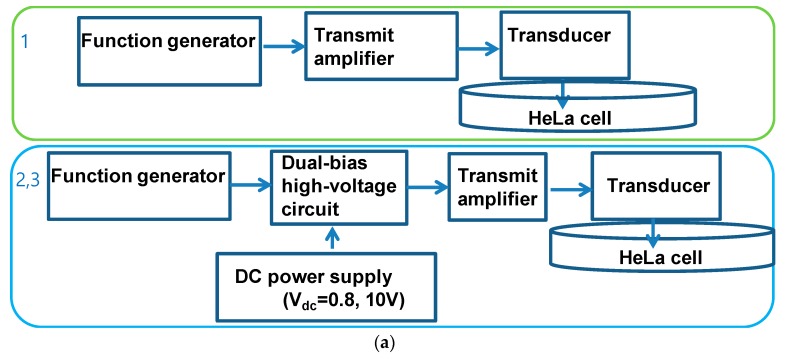

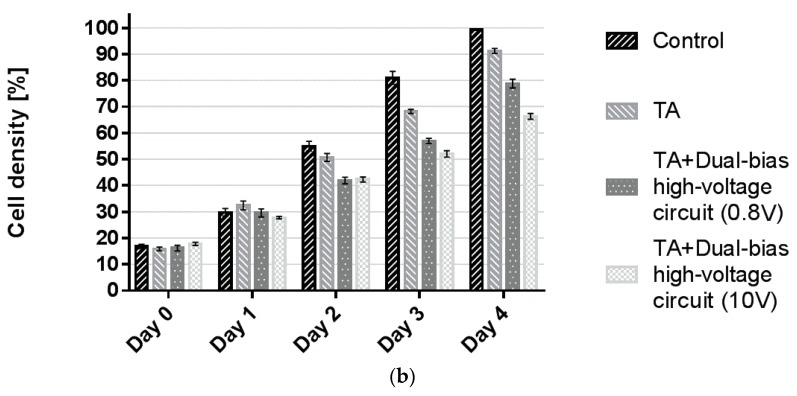
(**a**) Experimental setup of transmit amplifier only and transmit amplifier with dual-bias high-voltage circuit when 0.8 V and 10 V DC voltages were applied to the dual-bias high-voltage circuit. (**b**) Cell densities when using control group, TA (transmit amplifier) only, TA with dual-bias high-voltage circuit with 0.8 V DC, and TA with dual-bias high-voltage circuit with 10 V DC. * *p* < 0.05.

Figure 8a shows the experimental setup of the transmit amplifier only and transmit amplifier with dual-bias high-voltage circuit at 0.8 V and 10 VDC bias, as the harmonic distortion performances of the transmit amplifier with dual-bias high-voltage circuit are similar beyond 0.8 V DC bias voltages. This is because the operating bias voltage range of the primary transistor in the dual-bias high-voltage circuit is from 0.8 V to 20 V. For the 1st setup, 1-MHz and 10-cycle sinusoidal pulses generated from the function generator (AFG3252) were sent to the designed transmit amplifier to generate the ultrasound power to the 1.3-cm focused immersion ultrasound transducer (V303). For the 2nd and 3rd setups, 1-MHz and 10-cycle sinusoidal pulses generated from the function generator (AFG3252) were sent to the designed transmit amplifier with dual-bias high-voltage circuit to generate ultrasound power to the same transducer. For all experiments, the same power from the transmit amplifiers was generated to send electrical power to the ultrasound transducers. The dual-bias high-voltage circuit was controlled by the applied DC voltage from the power supply (E3631A). In the experiments, the same powers from the transmit amplifier with and without dual-bias high-voltage circuit were applied to the transducers to determine the effects of different harmonic distortions of the transmit amplifier.

As shown in Figure 8b, we confirm that the dual-bias high-voltage circuit can further help the transmit amplifier to reduce the cell density. The cell densities were measured quantitatively for following four consecutive days. The control group increased the cell density up to ~100% on day 4, but the ultrasonic signal induction groups (TA = 91.2 ± 0.8%, TA+Dual-bias high-voltage circuit (0.8 V) = 78.8 ± 1.7%, and TA+Dual-bias high-voltage circuit (10 V) = 66.3 ± 1.1%) demonstrated statistical significance compared to the control group (*p* < 0.05). On day 2, the cell density of the TA group failed to reveal a statistically significant decrement compared to the control group, unlike the other experimental groups. However, all experimental groups demonstrated a significant difference from day 3 to day 4. Because the operating bias voltage range of the primary transistor of the dual-bias high-voltage circuit are between 0.8 V and 20 V, the transmit amplifier integrated with the dual-bias high-voltage circuit was conducted beginning at 0.8 V. As shown in Figure 4d, the measured values of the HDs and THDs of the transmit amplifier integrated with the dual-bias high-voltage circuit are similar after 0.8 V. Beyond 0.8 V, the performances of the transmit amplifier integrated with the dual-bias high-voltage circuit are stabilized; therefore, the DC bias voltage of the dual-bias high-voltage circuit were selected to be 0.8 V and 10 V. 

## 4. Conclusions

A transmit amplifier with dual-bias high-voltage circuit was proposed to enhance the performances of an immersion ultrasound transducer because the dual-bias high-voltage circuit could boost the performances at the output voltages of the transmit amplifier. The performances of the transmit amplifier are crucial in the ultrasound system because the transmit amplifier triggers ultrasound transducers directly for therapeutic applications. Therefore, we need to use the dual-bias high-voltage circuit which can keep the stable DC bias voltages of the transmit amplifier such that we calculated and measured DC bias voltages under different time period for therapeutic applications. Calculated DC bias voltage of the transmit amplifier with and without dual-bias high-voltage circuit was 4.99 V and 4.0 V, respectively. However, measured DC bias voltages moved from 4.0 V to 2.75 V for transmit amplifier only and from 4.0 V to 3.92 V for transmit amplifier with dual-bias high-voltage circuit because unwanted heat affects DC bias voltages of the transmit amplifier. The DC bias voltages of the transmit amplifier integrated with dual-bias high-voltage circuit are provided by the voltage regulator with two variable resistors together with high-voltage transistor in the 2nd bias circuit. 

To evaluate the performances of immersion ultrasound transducers triggered by the transmit amplifier with and without the dual-bias high-voltage circuit, we performed typical pulse-echo measurements and demonstrated enhanced harmonic performances. All HD2, HD3, HD4, and THD values when using the transmit amplifier with dual-bias high-voltage circuit (HD2, HD3, HD4, and THD = –34.88 dB, –34.99 dB, –51.99 dB, and –32.02 dB, respectively at 10V DC bias voltage) were lower than those of the transmit amplifier only (HD2, HD3, HD4, and THD = –21.31 dB, –30.86 dB, –42.12 dB, and –20.96 dB, respectively). The designed dual-bias high-voltage circuit could stabilize the performances of the transmit amplifier with a wide DC bias voltage ranges by reducing the harmonic distortions of the transmit amplifier, thus generating cleaner echo signals. The control group without any ultrasonic induction increased the cell density up to ~100% on day 4, but the experimental groups with ultrasonic induction (TA = 91.2 ± 0.8%, TA+Dual-bias high-voltage circuit (0.8 V) = 78.8 ± 1.7% and TA+Dual-bias high-voltage circuit (10 V) = 66.3 ± 1.1%) demonstrated statistically significant cell density changes compared to the control group. Therefore, the therapeutic effects of HeLa cells using immersion ultrasound transducers triggered by the transmit amplifier with and without dual-bias high-voltage circuit were validated.

## Figures and Tables

**Table 1:** Measured DC bias voltages of the transmit amplifier with and without dual-bias high-voltage circuit.

Time	Transmit Amplifier		Transmit Amplifier Integrated with Dual-Bias High-Voltage Circuit	
	Calculated	Measured	Calculated	Measured
1 min	4.9 V	4.0 V	4.99 V	4.0 V
1 h	4.9 V	3.95 V	4.99 V	3.97 V
2 h	4.9 V	3.28 V	4.99 V	3.95 V
3 h	4.9 V	2.75 V	4.99 V	3.92 V
